# An investigation into mothers’ experiences of their children’s functional tic-like behaviour and tic attacks

**DOI:** 10.1371/journal.pone.0292742

**Published:** 2024-01-02

**Authors:** Amanda K. Ludlow, Seonaid Anderson, Sally Robinson, Tamsin Owen, Tammy Hedderly

**Affiliations:** 1 Department of Psychology, Sport and Geography, University of Hertfordshire, Hatfield, United Kingdom; 2 www.neuro-diverse.org, Durbuy, Belgium; 3 Southend, Essex and Thurrock Child and Adolescent Mental Health Services, North East London NHS Foundation Trust, London, United Kingdom; 4 TANDeM, Evelina London Children’s Hospital Guy’s and St Thomas’, London, United Kingdom; 5 Kings College London Faculty of Life Sciences and Medicine, London, United Kingdom; University of Sharjah, UNITED ARAB EMIRATES

## Abstract

**Objective:**

This is the first study to systematically explore the lived experiences of sudden and new onset of severe functional tics from the perspective of the mother’s experiences and describes their attempts to access support services in the United Kingdom.

**Method:**

Twenty-One mothers of young people aged between 12 to 17 years with functional tic-like behaviour (FTLB) took part in semi-structured interviews. Thematic analysis of the transcribed interviews revealed gaps and inconsistencies within the process of gaining access to professional services and a lack of support for the management of tics and functional tic-like movements, in addition to highlighting the impact it had on daily family life.

**Results:**

The themes generated included the occurrence and development of tics, the severity and intensity of symptoms, the psychological impact on the family and the need to make recommendations for a clear care pathway. Managing the impact of the FTLB and co-occurring conditions such as suicidal ideation and self-harm, as well as the physical and emotional trauma, commonly contributed to feelings of isolation and helplessness, which impacted negatively on the family’s ability to function and participate in society.

**Conclusions:**

The findings emphasize the urgent need to create a clear management pathway for those experiencing FTLB, including the need for more professionals with relevant knowledge, to improve the dialogue with families during the referral process, whilst prioritising the treatment of anxiety and other identified mental health concerns.

## Introduction

The recent coronavirus (COVID-19) pandemic led to a global health crisis and saw the United Kingdom (UK) government implementing extreme societal restrictions and social distancing. From March 2020 to April 2021, lock down restrictions included home confinement for many excepting essential activity, with school closures enforced from March-May 2020 and again in January 2021-May 2021. The transition from school learning to online learning at home was thought to affect an estimated 80–90% of young people in the UK [1]. During the pandemic and resulting lockdowns there has been a worsening of mental health in children and young people considered to be a direct effect of the pandemic [2–3]. There has also been a significant increase in incidence of tics and functional tic-like episodes in children and adolescents [4–5].

Tics have been described as an inability to suppress or an urge to perform, patterned, repetitive movements which can be sudden, rapid and include both non-rhythmic motor movements and vocalisations [6]. They are typically associated with Tourette Syndrome (TS) a neurodevelopmental disorder characterised by both motor and vocal tics occurring frequently for at least a year [7]. The DSM-5 (Diagnostic and Statistical Manual of Mental Disorders) and ICD-11 (International Statistical Classification of Diseases and Related Health Problems) also recognize that tics can appear as symptoms of other conditions, including in Unspecified Tic Disorder (8). Furthermore, when symptoms involve persistent motor or vocal tics, a diagnosis of Persistent (chronic) Motor or vocal Tic Disorder (symptoms more than a year), or a diagnosis of Provisional Tic Disorder (symptoms present for less than a year), is given [7, 8]. Tics can also be associated with atypical neurodevelopmental difficulties, for example specific deficits in cognition or variable mood problems. Only 10–15% of individuals with TS present exclusively with tics, with many individuals also presenting co-occurring conditions such as attention deficit hyperactivity disorder (ADHD), obsessive-compulsive behaviours or disorder (OCD), and/or autism spectrum disorder (ASD) [9].

During the pandemic, between 48–67% of individuals with a pre-existing tic disorder reported a worsening of their condition, often across multiple domains, which included, tics, hyperactivity, rage attacks, obsessions, and anxiety [10–11]. There were also high numbers of new referrals of children and young people showing tic-like behaviours within the subset of functional movement disorders (FMDs) [4,12]. FMDS are considered to sit within a broader category of functional neurologic symptom disorder (FND), previously termed conversion disorder, hysterical neurosis, somatoform disorder, dissociative disorder, and psychogenic disorder. Children and young people who show FND symptoms can present with motor symptoms that affect the limbs, voice production or the digestive tract; non-epileptic seizures; loss of sensory function; dizziness and balance difficulties and fainting episodes. They can also experience comorbid pain as well fatigue, nausea, disrupted sleep and changes in vision and/or cognitive function [13].

The increase of referrals of functional tic-like behaviour (FTLB) has largely been reported in females showing complex motor and vocal/phonic tics. The elaborate presentation in these acute cases, seen mainly in adolescence, have been identified as having a functional neurological disorder presenting as functional tics and tic-like attacks or functional collapses and faints. A ‘tic attack’ is a term that has been used to describe bouts of severe continuous, non-suppressible and disabling tic like episodes that can last from a few minutes to several hours. Tic attacks may be a mixture of intense episodes of typical tics combined with movements that resemble tics but seem to reflect the physical expression of severe anxiety or be functional in nature [14]. These episodes often include whole body writhing movements, muscle tensing and shaking sometimes resembling epileptic seizures, often resulting in a diagnosis of dissociative or non-epileptic seizures. They are distinct from the Tourettes phenotype, as tic-attacks do not follow the same pattern or phenomenology as Tourette related motor and phonic tics. The terms ‘tic-like behaviours’ or ‘functional tics’ or a ‘functional movement disorder’ may instead be used to describe these movements. These tic-like episodes have been described as painful and hard to manage [14] and, it is important to note that these young people show little or no response to the usual medications for more typical tics [5].

FTLB have been described prior to the Covid pandemic [15, 16], although these cases have previously represented only a small fraction of referrals to Tourette syndrome/tic disorder clinics [17]. However, during the pandemic there was a significant increase in the risk of having a FND during this period [18]. For example, in London 2019/2020, UK specialist tic clinics at each of two children’s hospitals were seeing four to six referrals for FTLB per year but during the 3-month period of November 2020 until January 2021, this had increased to three to four referrals per week [5]. While this approximates new referral cases to around 150–200 cases per year, this only reflects individuals who have managed to secure a referral to a specialist tertiary clinic, with the actual figures likely to be much higher.

Patients presenting with a FTLB can be distinguished from those with a Tourette related disorder, although this can be challenging even for experienced clinicians [19, 20]. The suddenness of onset of the movements, often occurring together with vocalisations can be a useful indicator, with functional tics typically occurring suddenly within hours and or on a specified day. Clinicians are debating how best to define the differences between the types of tic-like movements and tics [21]. The clinical course, atypical age of onset and type of tics can also be markers distinguishing functional tic-like behaviour from a tic disorder like TS [22]. For example, typically, childhood tics have an onset around 5 to 7 years and show a waxing and waning course of predominantly motor tics [23], whereas the functional motor and vocal tics found in the rapid onset group typically occur in adolescence and are usually more complex involving larger parts of the body and interfere more with voluntary actions, being both less suppressible and more suggestible [24]. However, recent research has cautioned about drawing any clear categorical distinctions between TS as opposed to functional tics, due to the high level of symptom overlap and co-existence of motor and vocal tics. Evidence also suggests that there exists a high probability of error in differentially diagnosing TS vs FND, and risks TS not being diagnosed when it should [25].

Interestingly, the new surge of FTLB appears more in females [26] compared to the higher incidence of tic disorders reported in males [27]. For example, in a retrospective review of 34 consecutive paediatric patients presenting with sudden onset tic-like movements, seen over 6 months, 94% of patients were female, with an average age of sudden onset or increase of tic-like movements of 13.7 years. Forty-four per cent had a previous diagnosis of tics, and 47% initially presented to an emergency department. Comorbid psychiatric and neurodevelopmental disorders were reported in 91% and 68% reporting anxiety [4]. Differentiation in levels of anxiety experienced by adolescent females may make this group particularly vulnerable, particularly if they are carrying an additional neurodevelopmental diagnosis [28].

In the context of the COVID-19 pandemic it has been suggested that the associated increase in screen time and use of social media platforms, may have contributed to the sudden increase in FTLB, resulting in this sometimes being described as “TikTok Tics” or suggested to be an expression of a “Mass Social Media Induced Illness” [29–30]. Some patients with FTLBs have described watching TS videos or posting videos of their movements on TikTok (www.tiktok.com). However, in a recent study of 185 Australian tic patients, only 18% had reported previous exposure to tics on social media when they developed symptoms [31]. This suggests that social media is unlikely to be the cause of the symptoms, but for some may serve as a trigger and/or exacerbate tic like movements, as viewing tics, albeit videos posted online or simply being around others who tic, can make a person’s condition worse if they have a propensity to tic, due in part to the suggestibility.

Heyman and colleagues [32] have suggested that the young people presenting with FTLB may form two distinct groups based on their presenting symptomology. The first group may represent explosive functional tic-like behaviours within a group with pre-existing Tics or Tourettes, whereas the second group is considered to comprise a group with new onset of tic-like disorder that appears functional in nature with no diagnosis of preceding tic disorder. There are similarities observed in both groups, including the higher risk of having an undiagnosed neurodevelopmental impairment, such as ASD, specific learning difficulties and attention deficit hyperactivity disorders (ADHD). Moreover, the precipitating factor for both groups is often anxiety (potentially in part COVID-19 related) and management for the functional tics can be similar for both groups.

Research to date has focussed largely on the symptomology of those with FTLB who have retrospectively presented at clinics, with still no firm consensus over the features for diagnosis nor evidence-based plans for treatment [33]. Despite widespread reports in the media and academic literature, no research has addressed the lived experiences of the families of young people with FTLB. This paper will present the first-hand parental experiences of living with FTLB in the young people they care for.

## Materials and methods

### Participants

A purposive sampling method was used to recruit parents of young people who had developed sudden tics within the period from beginning of the first UK lockdown to the following summer, where a variety of restrictions were still in place (23^rd^ March 2020 to June 2021). To recruit participants, information about the study and participation was shared in an advert via the Tourettes Action website and social media groups such as Twitter, Facebook and Instagram. Participants had to be parents of children and/or adolescents aged between 10–17 years old, who resided in the UK and whose child had developed sudden onset of tics and tic like movements during the pandemic. Anyone interested in taking part was asked to contact the first author via email where they were then provided a participant information sheet about the study. If after reading the participant information sheet, the parents still wanted to take part in the interview, they were directed to an online consent form and a time was arranged to do the interview. In total, 21 mothers aged from 37 to 53 years old (*Mean* = 43 years, *SD* 4.9 years) with a child aged between 12 and 17 years old (*Mean* = 13 years, *SD* 1.3years), 19 females and 2 males, were recruited to take part in the study. No fathers answered the survey. Families came from all areas of the United Kingdom including, Scotland, Wales, East, Southeast, Northwest and Southwest of England as well as the Midlands. No mothers who expressed an interest were excluded. Participant information is provided in Table 1. In the following accounts, names of people were changed to preserve anonymity.

**Table 1:** Participant demographics and a summary of their child’s history and development of FTLBs.

	Age	Child’s Gender and Age	Prior Diagnoses	Date of sudden onset	Length it started	Tics	Diagnosis given for tics	Premonitory Urges	Ability to Suppress	Self-Harm	In school	Health Professionals involvement to date
Linda	46	F 14	No -but referred for autism assessment	January 2021	2-days	Phrases or words that were sounds that were legs and arms. Collapsing	Previously diagnosed Functional Tics now considered conversion syndrome	Yes	Yes	No	No	GP CAMHS
Claire	39	F 12	Dyslexic Now on the ADHD pathway and suspected ASD traits	November 2020	1 month	Squeaking Whistling Clapping her hands hits out with her tics she gets stuck in positions where her arms or hands lock. Leg Paralysis	Diagnosed with Tourette Syndrome Suspected FND Having non epileptic seizure	Yes	Yes	No	Yes	Paediatrician Neurology
Nicola	40	F 12	ASD sensory processing disorder selective mutism. anxiety and depression	January 2021	Weeks	Motor/Vocal noises squeak rolling of tongue words phrases and swearing.	Tourette Syndrome	Yes	Yes	No	No	CAMHS
Wendy	45	M 12	No but older and younger sibling having neurodisability assessment On ASD pathway Mentions OCD traits Suspected autism in family (brother and nephew)	December 2020	2–3 weeks	odd words and sort of finding things mouth open fully and yeah that’s a kind of a motor tick and then he also has sort of a compulsive hand waving Stuck in movement	Not yet-wants to get ASD diagnosis first	Yes	Yes	Yes	Yes	GP Now awaiting Autism assessment
Debbie	53	M 15	Aspergers ADHD	October 2009	weeks	Blinking Rolling of eye Non-epileptic seizure Contorted face	PANDAS Conversion FND	Yes	Yes	Yes	Yes	GP CAMHS Private
Helen	41	F 12	Autism	September 2021	weeks	Jerky shoulder Head turn Bit of exhalation	No diagnosis sought suspected functional anxiety related tics	Not sure	Not sure	Yes	Yes	No Does not want to
Lucy	x	F 15	GAD OCD traits	October 2020	Days	Head tic Clap tic Yelp Drop in tics Chest hitting tics Swearing Drop in OCD related tics	Conversion disorder	Yes	Yes	Yes	Partly	GP Paediatrician CAMHS Psychiatrist
Beth	50	F 17	Hypomobile Social anxiety disorder Commutation difficulties (receptive) Chronic pain Complex PTSD Complex trauma	November 2020	hours	Throat clearing Cough	Complex motor tics Tourette Syndrome in 2020 Sudden onset November	Yes	Yes	Yes	no	Paediatrician CAMHS
Kate	xx	F 15	some anxiety but not diagnosed	May 2021	weekend	drop attacks or non-epileptic seizures Throwing Tics	Waiting on a diagnosis	Yes	Yes	Yes	Yes	CAMHS GP
Amber	37	F 15	Anxiety and undiagnosed ocd	October 2020	24 hours	vocal ones very loud and very offensive and the physical tics very large floor drop, hand claw	Anxiety -no tic clinic	Yes	No	Yes	Yes	Neurologist GP Paediatrician
Jessie	45	F 14		August 2020	Week	Motor-leg and head movements Noise and words	Tics	Yes	Yes	Yes	Yes	GP CAMHS Counselling Paediatrician
Natalie	53	F 14	Autism Anxiety	September 2021	days	Noises Eyes rolling Blinking really fast Throwing arms and Hitting squeaky noises biting clenching	Anxiety related tic- provisional tic disorder	Yes	Yes	Yes	Yes	GP Paediatrician Counselling
Ruth	50	F 14	No	March 2020	Days	Yelling names Hand and arms held in position Violent shaking Swearing Kicking	Tourette Syndrome	Yes	No	No	Yes	GP Paediatrician
Mel	47	F 15	Anxiety May show OCD and ASD traits	December 2020	Hours	Random Noises Shouting and Yelling unusual phrases Motor tics and the head Hitting herself in the face	Functional Tics	Yes	Yes	Yes	Yes	GP
Ally	45	F 14	Anxiety Panic Attacks Hypermobility Undergoing assessment for adhd and asd	April 2021	Slowly crept up over months	Neck tics Shoulder tics Whistling tics Vocal tics (words or sounds)	Functional Tics	yes	yes	yes	No	Therapist neurologist Private ADHD and ASD consultant
Rebecca	43	F 15	Absence seizures	November 2020	Hours	Stuttering, Wink Neck Movement Swearing	Tourette Syndrome	Yes	Yes	No	Yes	Paediatrician neurology Neurophysiology
Jane	52	Transgender M 15	Autism Dyslexia Anxiety OCD dissociative identity disorder Referred for ADHD pathway	November 2021	Months	Body shudders Swearing Neck and shoulder Leg tics Popping noise	Complex Tic disorder	Yes	Yes	Yes	Yes	Neurologist
Anne	48	F 15	social anxiety autism potential adhd traits	Summer 2020	48 hours	Swearing Motor tics Repeating things people had said, theme tunes. Whoops Whistles	Functional neurological disorder	Yes	Yes	no	Yes	GP Hospital had EEG and FMRI and was discharged Consultant
Shelly		F 13	ADHD Autism Anxiety	March 2021	A week	Head Twitch Random Words Punching Kicking	Functional tics	Yes	No	Yes	Yes	CAMHS Psychiatrist

### Procedure

The semi-structured interview schedule aimed to explore the child/family history and parent’s experiences of living with sudden onset functional tic disorders from initial onset, diagnosis, their impact and support. The interview included a family history of functional illness and the child’s physical and mental health. The interview schedule was developed based on an overview of the academic literature on FTLBs at the time and was also informed by the researchers accounts of their own clinical practices. The interview was further refined through discussions within the research team and with the mother of a child with FTLB, who was a personal contact of the research team and did not take part in actual study. The mother checked the relevance of the questions and appropriateness of the terminology used in the questions and participation adverts.

Key questions in the interview included:

- Can you describe how and when you first noticed your child showing signs of possible tics?
- Can you describe the movements- tics/type of tics/tic like episodes?
- Have you noticed any potential triggers that appear to exacerbate your child’s tics?
- Have you notice anything that helps the tics/tic like attacks?
- Can you tell me how the tics have impacted on your child’s behaviour (home, school, social life)
- What do you believe have been the main challenges of the tics for your child? (Probes- family)
- What support and professional help has been provided to you?

Due to the social distancing measures in place at the time of the research, interviews were conducted through an online platform, and lasted between 33 minutes and 1 hour 7 minutes. Following the interview, participants were given the opportunity to share further information, thanked for their time and were given a debrief sheet.

#### Ethics

Ethical approval was granted by the institution’s ethics committee (protocol number: aLMS/SF/UH/0461D1(1). Participants received and signed a consent form informing them how their confidentiality would be maintained, and data used in a publication. Interviews were carried out and recorded by the first author, who had 15 years’ experience of qualitative research methods. The completed interviews were downloaded immediately, saved under a unique code and pseudonyms applied. Following transcription by the lead author, video recordings were deleted.

### Data analysis

Inductive thematic analysis was used to allow for an understanding and interpretation of participants’ subjective experience [33]. The lead author read and listened to the data several times to ensure familiarity; and preliminary themes, comments, and interpretations. Transcripts were analysed manually, primarily on a semantic level (i.e., ideas and explanations explicitly communicated by the participants were prioritised). The analytic process involved immersion in the data, reading, reflecting, questioning, imagining, wondering, writing, retreating, returning. Common themes were collated across transcripts and analysed for similarities, creating subordinate themes across the data. Through discussion within the research team, subordinate themes were refined and analysed, and further grouped under superordinate themes. The sample size was appropriate for thematic analysis, with recommended sample sizes of between 6 to 16 interviews, to achieve data saturation (see 34; for a review of the saturation debate). This is appropriate for the inductive participant lead content coding that was required for this research project.

### Data credibility and trustworthiness

To ensure data credibility, rigor and trustworthiness, we adopted the framework proposed by [35] and [36]. This included in-method triangulation, member reflections and audit trail, as well as providing an opportunity for participants to view the final write-up of themes and relevant quotations for comment. The research team was composed of clinicians specialising in movement disorders, with their services at the time of the study experiencing an increase in referrals for FTLBs. The design of the study was also informed by members of the FTLBs community, with a mother of a child showing FTLBs, who did not take part in the actual study, kindly reviewing the questions for appropriateness and relevance. We adopted the term tics to reflect the mothers’ experiences and favoured terminology.

## Results

Analysis of the 21 semi-structured interviews resulted in 13 subthemes grouped into 4 themes and outlined in Table 2: The occurrence and development of sudden onset of tics; The severity and intensity of the symptoms; Psychological impact on the families, the need to have a clear pathway forward.

**Table 2:** Themes and subthemes from the interviews.

*Themes*	*Subthemes*
The occurrence and development of sudden onset of tics	The shocking speed and severity to which tics appeared An initial anxiety provoking event Tics an outlet for anxiety Common triggers for the tics
The severity and intensity of the symptoms	Physical impact Emotional torture Lack of access to education
Psychological impact on the family	All encompassing Loss of normal life Helplessness
The need to have a clear pathway forward	Frustration over lack of professional understanding and dismissiveness The desire to have a clear pathway to care Importance of Parental Advocacy

### The occurrence and development of sudden onset tics

All mothers discussed the negative and psychological impact of managing their child’s comorbid mental health.

#### The shocking speed and severity at which the tics appeared

Many of the mothers were shocked by the speed for which the tics suddenly occurred, which was further magnified by their severity and intensity.

It was the week before half term, so she’d gone back after the lockdown to school in September. She hadn’t been to school previous to that since March, and she came home from school one day, the week before half term and it was like a light switch. We had never seen anything before that, but within 24 hours she was ticking constantly with movements and silly type noises…it was completely debilitating. She could not move from the way the tics were, violent and very big and she couldn’t speak because everything that came out of her mouth was uncontrolled by her. It was that quick.

(Amber)

She went back to school and from then it just sort of spiralled. So, there were lots of new tics coming all the time. One day she had some leg movements and then with it the next day she started with some head movements and jerking movements. So, over the space of a week she went from nothing to loads of different tics. It was very quick.

(Jessie)

All of the mothers believed the tics to wax and wane over time, however in the initial stages many of the mothers reported them to be unremitting.

They don’t stop okay, so their constantly there. She doesn’t tic only once she has properly gone to sleep. You can hear when she’s dropping off. You can hear them reducing.

(Linda)

I think I ended up calling an ambulance because she couldn’t eat, she couldn’t drink, she couldn’t stop through the ticking being that bad, because I didn’t really know what to do. I hadn’t seen it before.

(Claire)

#### An initial anxiety provoking event

All parents were able to give an account of the timing and run up of events from when tics first appeared. Similarly, all parents highlighted their children to have a history of anxiety, and most were able to link the start of the tics to a specific anxiety/trauma provoking situation. For example, Lucy stated “There was a clear pathway to her anxiety increasing to a significant event that triggered the tics and then it just went downhill.”

Kate described her child as experiencing a dissociation episode after a heated argument with friends, which led to her child showing “not quite right behaviour over weekend*”* with the tics appearing on the Monday. Whereas Wendy described an emergency evacuation back to the UK following the Covid outbreak, having moved countries. For others it appeared school related.

She already suffered anxiety in general. During the pandemic she left the primary to start secondary school, so that was difficult enough doing that transition. When she was in her last year of primary school, when the lockdown first started, they were doing all on teams and she really struggled with that.

(Shelly)

Literally, we can pinpoint the minute she was sitting on a team lesson in her bedroom. She has always been one of those that worries about how people perceive her, and online video learning was just the final straw, because it has started two weeks before this all started. She was clearly worried about how she was going to be perceived on screen. Russian roulette of who was going to be asked to put their camera on and answer a question and things like that.

(Linda)

An online session on abuse which is her background, but they hadn’t warned her. At 8 o’clock that night she had a 9-hour tic attack which just did not stop.

(Beth)

I suspect it was after the school had started and so obviously that’s a big transition into secondary school

(Helen)

Worrying about loved ones appeared to be another trigger:

Lost both my parents to covid last year suddenly. There was very little warning for it, and he said he thinks that was the trigger.

(Jane)

Husband crashed his bike and broke the ball off the top of his femur. That was quite traumatic because we were away from home, and he was hospitalized. We couldn’t go and see him because of covid and she was very upset about that.

(Natalie)

### Tics as an outlet for the anxiety

All mothers were clear there was a relationship between anxiety and tics.

If there’s something that’s causing anxiety coming up that will cause the tic

(Jessie)

She was really worried about covid due to her pain. Me, my husband and her sister all had it, and then my husband was just beginning chemo so the anxiety of all of that caused a tic attack.

(Beth)

I mean my personal feeling on it is, that if he’s getting help to manage his anxiety, then the tics will be part of that. I hadn’t really occurred to me to sort of address them as a separate thing you know. I am hopeful that they will suppress, and we would see them disappear again in time if the anxiety is controlled.

(Debbie)

Definitely anxiety is a trigger. My dad got diagnosed with terminal cancer in the summer and a lot of new tics developed. She also tics more in classes she doesn’t feel so academically able in

(Louisa).

Many of mothers reflected on the fact the tics, rather than being a completely new phenomena, may be a new manifestation of their child’s existing anxiety.

Her psychologist once said, maybe the tics have come to set you free. And I think you know that’s a little bit about facing some anxiety.

(Linda)

It was an expression of a feeling that she’d always had, you know that what was a difficult time of day. For her it was just coming out in a different way now, through these kinds of physical tics.

(Helen)

The tics were even considered by Helen, a mother of a child with comorbid autism, as being an unconscious way of helping her child to understand her own emotions better “It does feel like a physical external manifestation of an internal sensation and so, in that sense, I wonder if it’s helping her understand her own feelings.”

#### Common triggers for the tics

Outside of anxiety, other common triggers were fatigue. “

Definitely tiredness. If she gets tired, she has more tics or if she’s stressed or worried about something

(Jessie)

The kind of tiredness that comes from the school day and the kind of fluctuations through the day when she’s hungry, or if she’s anxious about something. It’s just now being expressed through tics, when previously it was expressed in other ways.

(Helen)

If she’s tired, she tics. If she hears sudden loud noises, they will make her tic. Crowds and crowded places because they increase her anxiety, that will make her tic.

(Natalie)

Sensory issues were also raised by some mothers. For example, changes in room temperature were raised as being a trigger by both Rebecca and Flo respectively: “I think things like the cold and the hunger that she did find the triggers” “When there is any type of extreme. So, when she is hot she tics more, when she is cold she tics more. When she is hungry and when she is tired, she tics more.” Others highlighted the school environment to be another trigger.

She has a lot of sensory issues that we were not really aware of before. When she is really cold, that makes it worse. School makes it worse because she finds the classroom is really really busy, noisy and bright. She finds it all really difficult.

(Ally)

Worse at school than they were at home, because obviously home is the sacred space

(Rebecca)

Interestingly, all mothers showed awareness of TikTok, though only half of the young people were reported to be known as regular users to their mother’s knowledge. For example, Louisa stated “I now know she had gone on social media and followed some tic accounts because she was worried.” In all cases, parents acknowledged the platform made the tics worse, as did watching or being around others with tics. All were clear, like Ruth that it was not the cause “She’s uses tick tok she does, but that is the trigger not a cause.*”*

### The severity and intensity of the symptoms

The emotional and physical pain of the tics for their child resonated strongly in all the parental accounts.

#### Physical impact

The inability to control the tics was leaving many of the children in physical pain, with Lucy commenting her child’s hitting tics meant she was continually black-and-blue bruised all over, which was reiterated by Ally “She hits her own head. this is the worse one as she has injured herself quite a bit”

There were times where I had to sit next to her and hold her hands for about three hours at a time, because otherwise, she would just be hitting herself.

(Amber)

Ella had what I can only describe it as a tic attack. And we ended up at A and E and it took them two different lots of sedation to finally calm her down. She was punching herself in the head, she was biting herself. And me and her eldest sister were having to physically restrain her, and she was distraught. She was really crying.

(Shelly)

She was waking in the night have a tic attack and it looked like she was having a seizure…the pain going down her spine was just incredible.

(Anne)

Some of the mothers highted the physical nature of tics went beyond hitting themselves.

He has broken windows smashing fists

(Jane)

She had a strangling tic for a while where she would try and strangle herself.

(Louisa)

Several of her tics are ocd tics and they are all the dangerous ones. The urge to touch a boiling kettle, the urge to put her hands in boiling water. She had an ocd tic where she had the urge to smash the window as hard as physically possible. Trying to open car doors as she was going along. Swearing at people in the street….she had a tic which made her grab bleach bottles and want to drink the bleach.

(Lucy)

The inability to breath and paralysis was also mentioned by four of the mothers.

Her throat closes and she couldn’t breathe and this could go on for 30 to 40 seconds and after an 1 hour I phoned 111, never seen anything like that so I didn’t know what to do. Because of her inability to breathe I called an ambulance, and she was taken into hospital.

(Beth).

He had phases, where he was holding his breath constantly and his legs didn’t work, arms didn’t work, voice didn’t work, and he ended up in hospital a month later because nothing was working.

(Wendy)

About 14 minutes later she came down clearly hadn’t been asleep and she has been hitting herself like this in bed, and she couldn’t stop. She couldn’t make her legs move, so she could come and tell us. So, she came down very, very distressed.

(Linda)

She had a new teacher, and she was nervous about that and managed to suppress her tics through the lesson. Afterwards she went to the intervention room and ended up paralysed for the next three hours unable to go to her next class.

(Louisa)

Self-injury also appeared as a way of coping with the distress the tics were causing.

She was so self-conscious and aware that people were watching her, but for her releasing the anxiety through hurting herself was preferable.

(Natalie).

Quite a lot of self-harming such as scratching his face for a while. I managed to get some cotton gloves for him, and then the ticking took them off before he scratches the surface. So, it’s like this little demon inside him was circumventing anything we put in place to kind of make it okay, to find another way to be upsetting.

(Jane)

#### Emotional torture

The physical tics are often noted as being debilitating and more obvious to others. However, the internal symptoms were described as equally troubling to note, and to be challenging for mothers having to watch their children’s internal struggles. For example,

He came home one day toward the end of that week and lay his head on my lap, as I was sitting on the floor, and his whole body was going. And he just said mom, I can’t live like this, there must be something I can take?

(Jane)

She would be ticking every day at the beginning of the cycle, she would not necessarily harm for one or two days, but then she would be harming, and it would get worse as the tics would get more severe. Then at the end of that cycle, she would just collapse. She would be just curled up in my arms saying I want this to stop, when is this going to stop? I don’t want to feel like this any longer.

(Natalie)

Every time the phone goes, I think it’s going to be school, and I really prayed so hard, but the tics are progressing and worse with time. It’s now making her really depressed and miserable with it.

(Kate)

When the tics first started, during a month of it she got really depressed and tearful. In the middle of one attack, she had a tic where she was screaming, I want to kill myself.

(Lucy)

With Nicola mentioning the tics being the final catalyst to cause her daughter to collapse. “The tics were like the final straw in terms of her mental well-being. That is when we kind of reached suicidal levels”

#### Lack of access to education

Mel highlighted schooling being unattainable “She’s missed lots of school and even when she goes to school, she’s not really accessing lesson” with many of the parents also commenting on their child’s lack of ability to cope in school as a direct consequence of the tics.” Similar sentiments were echoed by other mothers.

She is probably on about 55–60% attendance something like that. I would say most of the time it is due to anxiety, but it’s all very very closely linked because the anxiety is worse because of how people treat the tics at school. I think the tics developed out of an anxiety thing. It’s like a vicious circle.

(Ally).

When he was having the ones with the arms and the legs, and they were so severe that actually we found just walking into school was too difficult.

(Debbie)

She loves school. Absolutely loves school and wants to be there, but she’s unable to stay in classes. And so, she has got a one to one at school because safety wise she has so many seizures and her tics can become so bad safety wise. They must have somebody with her all the time.

(Claire)

For some this had left them to the decision to remove their child from the school. For example, Beth and Debbie both removed their child.

She came out of school November 2019 because of anxiety and constant pain.

(Beth)

We had to remove him from his school because it was an unsuitable placement, he self-harmed…since he started at residential school where he has speech and language to class size of four to six people. His tics are virtually gone.

(Debbie)

Other parents highlighted a more positive experience of dealing with the tics when the schools were better able to support them.

She has some issues with teachers who just didn’t understand what tics were. But they were dealt with very quickly and very synthetically and people were educated and so, even in that respect I don’t think we could have asked anymore from school…. school were incredibly supportive and have Friendship Group they call themselves the tics army and they literally enveloped the inner kind of protective bubble, and so she never tried to hide the fact she had tics.

(Rebecca)

Schools have put stuff in place which basically mainly involves getting her away from lots of people and being able to go to a quiet space, and stuff like that helps her tics.

(Ally)

In some cases, schools were reported to have helped the families manage the tics by supporting home learning.

School very quickly realized that the best place to say tics free was not sort of trying to get her back into school at this moment and sent her off to a team called the flexible learning team, who are a specialist team for children who are out of school with medical needs.

(Linda)

### Psychological impact on the family

All mothers drew attention to the significant impact that living with a child with sudden onset of tics had had for all members of the family.

#### All-encompassing

Amber and Lucy, both mentioned it impact, “It’s been a total change of her life and ours” “It’s just how life changing it has been and how much it affected everybody in the family, highlighting the the all-consuming impact the tics have had on so many different aspects.

It clipped all our wings. There was not a lot we could do anymore because of the tics. We cancelled holidays. We didn’t have anyone around.

(Anne)

Causing stress and worry. Worry about her injuring herself and not going to school anymore, and financially, as we have spent a lot of money on just her with money on diagnoses and therapy.

(Beth)

For others this was noted by the complete change to their interactions with their child

It’s more than just physical movements, though it’s like almost like a complete personality change.

(Linda)

The tics started really quite minor, and then it has built up to now. I can’t now leave her on her own.

(Shelly)

#### Loss of everyday life

For most of the parents, some of the most difficult consequences of the tics came from the loss of seeing their child doing day to day activities.

In terms of impact, I think, just the fact that she went from being completely normal, you know social sweet hearted active girl, and she just literally in the space of 24 hours was incapable of doing that. It was not a lack of willingness. It was a lack of actually being able physically to do those things.

(Rebecca)

It’s really debilitating and it’s really hard work and she’s really tired afterwards, and she just wants to be normal and get on with it, and yet she can’t.

(Kate)

It was like having a toddler again. So, all that danger spotting you spot with a toddler you would have to do with her. So, dinner time there was like throwing food. Throwing knives, pouring drinks over her head. Just stuff that made everyday tasks really really difficult.

(Lucy)

It’s just the interruption to normal life. She used to be busy all the time academically and socially

(Flo)

Mothers commented on things that previously had given their child so much enjoyment suddenly being taken away.

She does a lot of ballet dancing, and she went from dancing on point for four hours a week to not being able to lift her feet off the ground in the space of a week.

(Rebecca)

It made her really upset…her favourite dinner, which she was really looking forward to eating, and she would just throw it across the room, and it would really devastate her. To be so out of control of her own body.

(Lucy)

She was rejected by her friendship group at school.

(Louisa)

For months she didn’t pick up her cello. She said she was because she was worried that she would throw it across the room

(Flo)

For some children the tics impacted on their ability to leave the house.

She doesn’t go out. If she goes out to a shop with me, she has grabbed things off the shelf and has thrown them or hits herself or drops to the floor, so there is I think, a lot of embarrassment over that. She doesn’t see friends very much at all.

(Amber)

Until recently she didn’t want to go out, not even for a walk around the block in case of her tics

(Anne)

She doesn’t want to leave the house. We have to absolutely force her out, basically, and we have to give her plenty of notice. We have to tell her exactly where we’re going and how long it’s going to take…… she just doesn’t want to see anybody. She doesn’t want to go out.

(Natalie)

#### Helplessness

All mothers highlighted a sense of helplessness in being able to help their child.

It’s really hard as a parent and not be able to really do anything to help and to this like feeling of helplessness

(Jessie)

So distressing not being able. To tell her when it’s going to stop or do anything other than just put my arms around her and tell her story. It’s just the helplessness that’s the hardest thing.

(Natalie)

It was just devastating to her and happened so fast and so out of the blue, and suddenly we realized we were dealing with a completely different situation to what we thought we were dealing with…we were just so heartbroken for her and so powerless to help her.

(Rebecca)

Sometimes I do feel very alone. It’s just hard. I’ve got a really supportive partner. He’s fantastic. But for me, I just find it really difficult sometimes.

(Ruth)

My mental health is in tatters because there is no support. Because it is horrific, knowing what she needs is early intervention and help

(Louisa)

### The need to have a clear pathway forward

#### Frustration and anger over lack of professional understanding and dismissiveness

All mothers expressed frustration over the lack of professional knowledge of sudden onset of tics and even Tourette syndrome as well as a distinct lack of support offered.

We are completely on our own, because the medical people that we’ve spoken to don’t have experience of it. We’ve been feeling around to try and find somebody who recognizes it. Just somebody to look at her and say, yes, this this is what’s happening to your daughter. Not necessarily be able to tell us how long it’s going to last, or whether it will ever go away. But just somebody to recognize it.

(Natalie)

Something’s terrible going on, and nobody wants to help, no one even knows. Even throwing money at it, we can’t get any help. So, it would just be having someone who’s given us the options and then listen and say well you know she’ll be all right or whatever.

(Kate)

Some mothers felt their child was being dismissed due to their anxiety, as highlighted by Shelly ‘I think the biggest thing for us is not feeling believed.’ Many parents expressed a desire for professionals to take their situation seriously.

They think she’s putting it on. That it’s not a real illness

(Ruth)

Just to be told, oh she is probably anxious, off you go and they said don’t come back. Because I kept ringing to say this has happened now. And they were saying, well, we can’t really help you. You just have to get on with it

(Jessie)

She didn’t have control over the treatments. She didn’t have control over what people were saying about her. She was absolutely furious that they were trying to put the whole thing down to Tik Tok.

(Rebecca)

Get your child’s anxiety treated and it being taking seriously because anxiety can sometimes be written off or seen as something that is not such a big deal.

(Lucy)

One mother reported a feeling of complete lack of support and empathic understanding by health professionals

“they said, do you think you’re going to kill yourself.” And she said “no, why would I do that?” and they went, “well if you’re not going to kill yourself there’s not really anything we can do”. And literally, I think half my chin fell on the floor at that point. I just said, “apart from the fact that this is wholly inappropriate, but what you’re telling us is that because she’s actually not at the point of wanting to commit suicide as a 13-year-old girl, you are doing absolutely nothing to help her cope with this complete change physically and mentally that she was going through”, and they said “no.”

(Rebecca)

#### The desire to have a clear pathway to care

A lack of clear direction of where to seek support was also reported by all the mothers and for most this was presented as reflecting a sense of frustration. With many mothers such as Jane highlighting the need for some practical solutions, “just some really concrete stuff, to clear signposting.” For other mothers it was having clarity as to where to turn to for help.

A clear diagnosis or a certain pathway to at least follow. We’ve not got a pathway

(Ruth)

There was no pathway, no reassurance, nobody who could say “Okay this can be a progression from the tics” and “this is what you can do about it, or look at these charities to help”

(Kate)

If there was a pathway whereby child presents itself, and then they go “okay first thing we’re going to do is this this and this, once all that’s done, we will then look at”

(Rebecca)

I think what it would have helped is certainly to have had a CAMHS service, who could have helped us

(Debbie)

Parents also expressed desire for a clear pathway with access to medical professionals at the start who had an actual understanding of tic disorders.

Having access to medical professionals in a timely way, who actually understood and had that specialism

(Debbie)

A professional. Someone who understood about tics or someone who would listen at the beginning

(Ruth)

To go to the doctor and they know what tics are and what Tourettes is, as they don’t know. I would like to have gone to the doctor and them to have given me some support. If I had been supported, we would have been a lot better off

(Ally)

The psychoeducation sessions on functional tics that were offered were pointless, as having lived with it for a year, it was kind of pitched at someone who had just had it in the first month

(Lucy)

For others, this was treating it as a mental health condition rather than neurological. To encourage her to treat her mental health as priority (Amber)

The only way we can stop them is to delve deep into all those underlying anxieties and really help her to kind of unlock them and maybe then gradually as we unlock all of that, the tics will start to go away

(Shelly)

A simple request for some reassurance was also noted.

Reducing the uncertainty about whether what you’re doing is right

(Linda)

I would like to be a community with other parents. So, the parents can reassure you

(Shelly)

Talk about it with anyone who knows anything about it so that you are not on your own

(Flo)

#### Importance of parental advocacy

Many mothers reported a need for persistence in the fight for their child, with many feeling that they were be often dismissed by professionals.

The psychiatrist has been fairly dismissive of the tics and it’s only through my absolute persistence and then kind of moaning at them

(Shelly)

Pushing for medical help to check, because you don’t know they can’t just say it’s anxiety at the first appointment and leave it at that.

(Jessie)

With mothers also expressed the need to not be afraid and to be completely unapologetic in seeking support for their child.

Just really fight for what you want, what you believe in and really push: go with your gut instinct.

(Ruth)

You have to just bloody push … you know you can’t leave your child like this because you know it’s never going to end.

(Lucy)

I would say hit harder than I did because I just thought it would go away. So, I would read about it. Try and look at sensible websites about it. I would speak to your GP but not be fobbed off, because that is what they do. I would speak to school about it and. Speak to your child about it.

(Kate)

Trying to arm yourself with information, good information written by doctors and written by healthcare professionals is important. Trying to keep in a constant dialogue with your young person. Trying to help them to be as happy and safe as they can be.

(Mel)

## Discussion

The literature surrounding sudden onset tic cases has tended to focus on identifying the development and function of FTLB [e.g. 10]. This has highlighted the complexities of diagnosis, due to core differences in symptom presentation and onset compared to the known associated Tourette syndrome phenotype [22, 25]. No literature to date had addressed the families’ experience, therefore, this study aimed to explore parents accounts of living with a young person with FTLB. Only mothers answered the survey. Qualitative analysis revealed that mothers reported a sense of aloneness and helplessness in how best to support their child with managing this health condition. This appeared to reflect a combination of difficulties gaining access to health professionals and an exacerbation of the functional tics by anxiety. Many parents felt that there were dismissed due to a lack of knowledge about the condition by professionals and a failure to appreciate the severity of the presenting symptoms. Moreover, mothers reported a high level of physical and emotional impact due to the FTLB’s, with symptoms affecting both the mothers and young person’s wellbeing and daily functioning.

Clinicians and researchers alike have speculated that increase in FTLB seen during the COVID-19 lockdown was caused in part by the pandemic [5]; with changes occurring in structure and routine, increased ‘screen time’, use of social media and pandemic-related stress all considered to have acted as triggers in vulnerable young people [37,12]. This was verified in this study, with all young people experiencing functional tics during and after the first lockdown, with most reported to have experienced the onset of symptoms over a short period of hours to days, with many able to give a precise date of onset. Importantly, all young people reported a history of anxiety, with some previously diagnosed with anxiety disorders (e.g. social anxiety disorder) and others reported to exhibit a high level of arousal and anxiety in daily life. This study supports the view that societal stressors/trauma contribute to the onset of FLTB.

Since the pandemic, it is has become clear that many of the FTLBs remain and there are still many cases of adolescents with FTLBs being referred for clinical assessment. The increased visibility and interest surrounding tics and tic-like symptoms has, in part, been led by the increase in the use of social media platforms (e.g., TikTok, Instagram), where some parallels can be drawn from those experiencing sudden onset of tic-like behaviours with the presentations of those being viewed on social media [38]. Given the time adolescents spend on social and media and internet, exposure to such content has increased [39]. However, there exists little empirical support for notion of a direct association between social media use and developing FTLBs, and as the mothers’ accounts highlight, there is clear need for researchers and clinicians not to assert unproven conclusions on this complex phenomenon. Reducing the stigma would lead to better understanding, care and advocacy for individuals experiencing these complex and life changing symptoms.

The mothers’ accounts provided support for the suggestion by Heyman and colleagues [32] that some young people who are showing FTLB, may also have an undiagnosed tic disorder. For example, it has been argued that stress may unmask symptoms in those who have an underlying genetic predisposition to tics. This was the case of ‘Ally’ who had recently been given a Tourette syndrome diagnosis herself and suspected that her daughter probably had the same condition. Similarly, in the current study there were five young people who already had a Tourette syndrome diagnosis but were also now exhibiting tic-like attacks. This group appears to be characterised by more explosive functional tic-like behaviours. In comparison, there may exist another group who show a new onset tic-like disorder that is functional in nature. Furthermore, in line with other cases reported in the literature [34], all the children were more vulnerable to anxiety and/or underlying neurodevelopmental or emotional difficulties to the point of becoming overwhelming.

There was consistency in the mother’s accounts regarding the triggers to the functional tics, with fatigue, anxiety, stress and lack of sleep all identified as being common. This is similar to that reported for children who present with functional tics in the context of a Tourette syndrome diagnosis [14]. Importantly, exposure to sensory stimuli was also suggested by all the mothers as being an important trigger of functional episodes, consistent with a previous study where hypersensitivity to external sensory stimuli (i.e., visual, tactile, auditory, motor) was linked to the sudden onset of functional movement disorders in children [36]. Therefore, information on sensory processing could be important in furthering the understanding on the potential drivers of FTLB and how these factors relate to the high levels of anxiety experienced by children and young people.

Geroin and colleagues [40] proposed a “predictive processing” explanatory model of FND, which highlighted that exposure to sensory information alters expectations and attentional focus. Triggering events are thought to function at a level of unconscious or automatic learning, that recreates the abnormal attention focus on repetition of event. This is akin to what has been described with panic autonomic arousal symptoms and functional seizures [41], as well as functional drop attacks and sudden falls that occur without warning, or which involve a loss of consciousness [42;14]. According to the predictive processing account, sensory responses reflect a hypervigilance that may have arisen following a traumatic or stressful event, with these responses (i.e. in these cases the functional tics) being reinforced through classical conditioning processes, such that is becomes the predominant response in future in stressful and/or panic inducing situations. In the case of the young person’s often debilitating anxiety, the tics may have occurred to relieve or avoid triggering sensations or thoughts [43]. The potential relationship between sensory, physical and emotional experiences was raised by mothers in the study, who considered the tics to be an outlet for their children’s existing anxiety.

The school environment was frequently reported as a factor that increased FTLB. For example, the transition to online sessions along with the pressure to perform during lockdown, was considered by some of the mothers to have been a possible trigger, while for others it was returning to school after lockdown that had contributed to the worsening of symptoms. This finding is consistent with previous research [44]. There was also a high level of school avoidance reported and the predominant view of mothers was that the sudden increase in expectations and comparisons by others (e.g. peers, teachers) had led to an increase in symptoms of anxiety, with children avoiding school due to requiring a greater level of personalised control within their environments.

It has been suggested that females compared to males are more likely to adopt avoidance-based strategies when faced with anxiety inducing situations [45]. Avoidance has also been identified as a coping strategy used by females with autism [46], with removal of environmental and sensory pressures reported by adolescent females with autism to support better emotional regulation [47]. Where schools had adopted a combination of ‘planned avoidance’ through flexible timetabling or home-learning, mothers reported their children to be coping better. For the young people attending school, a number were reported to use emotionally and cognitively demanding strategies to suppress functional tics during the school day to avoid stigma, which often resulted in extreme fatigue and tic-like attacks later in the day, as well as increasing anxiety overall.

A key theme reported by parents in this current study was the feeling of a lack of trust in the system. The views and struggles these parent’s experienced accessing professional support for their children highlights the need for clear care pathways in which children can be signposted to help so that professional input can be provided. Primary care professionals could benefit from the provision of specialist training to consider the differential diagnosis of tics and functional tics, as well as having a role to provide resources to support psychoeducation about management strategies at home and school. Increasing access to local Child and Adolescent Mental Health Services (CAMHS) with provision of evidence-based interventions for any presenting mental concerns is also required, as several of the young people in the current study were reported to be actively engaging in self-harm behaviours and showing suicidal ideation. Diagnostic shadowing, whereby the healthcare professional assumes that the clinical manifestation of physical illness was due to their existing anxiety may also risk the young person going misdiagnosed and undertreated, [48] which highlights the need for partnership working between paediatric and mental health services.

It is important to note some of the limitations to the current study. As with all self-selected research samples, the degree to which findings can be generalised to a wider population needs to be treated with caution, particularly given the limited available literature on FTLBs to draw comparisons. Further, everyone in the sample had accessed some NHS services, with some also seeking private health care. However, further studies are needed to understand the views of those who may not have reached out for support, those who have been successful in accessing NHS support that has met their needs and those of other traditionally ‘hard-to-reach’ communities, such as black and minority ethnic groups and those at risk of health inequalities. Equally, while FTLB has been shown to be more common in females than males [32], some studies have suggested that there is a more equal gender distribution, with one of the most notable social influencers on Tik Tok being a German male [20]. Whilst our sample mirrors previous research in reflecting a female dominance for FTLB, this must be interpreted with care to prevent introducing or reinforcing existing gender-related biases [25]. It is well documented that females with TS are more likely to show tics that are more complex and have a later onset. The tics are also likely to show greater functional impact, worsen with age and for many females with TS may co-occur with anxiety and mood disorder [26]. Therefore, future studies need to address how presentation may vary across genders, in relation to sex-based characteristics but also gender identity.

## Conclusion

This is the first study directly exploring the experiences of mothers of young people with FTLB, with rigorous, in-depth thematic analysis reflecting the context and experiences of this group. This study provides further support for the view that the FTLB can be distinguished from Tourette related tics by the acute timing of symptom onset, clinical phenomenology of the movements and by analysis of the wider bio-psycho-social symptom presentations. However, this can be challenging especially when both TS and functional symptoms co-exist. While there is clearly some overlap with wider FND symptoms, the optimal name or label being given to those showing functional tic-like behaviours is still undefined, as reflected by the varied diagnoses given to the young people in the current study. Questions also remain regarding the longitudinal nature and likely prognosis of children and young people with FTLB, with a need to identify the individual, systemic, environmental, social and emotional factors that might contribute to symptom presentation, maintenance and longer-term implications. This would help inform the development of evidence-based psychological treatments for use when necessary. Further guidance on symptom management at home and school is also likely to support improvements in the wider general well-being and quality of life for these children and their families. This is planned future work within our collaborative networks.
